# Central-place foraging poses variable constraints year-round in a neotropical migrant

**DOI:** 10.1186/s40462-022-00337-2

**Published:** 2022-09-20

**Authors:** Kristen M. Lalla, Kevin C. Fraser, Barbara Frei, Jason D. Fischer, Joe Siegrist, James D. Ray, Mario Cohn-Haft, Kyle H. Elliott

**Affiliations:** 1 Department of Natural Resource Sciences, McGill University, 21 111 Lakeshore, Sainte-Anne-de-Bellevue, H9X 3V9 Canada; 2 Department of Biological Sciences, University of Manitoba, Winnipeg, Canada; 3 Wildlife Research Division, Environment and Climate Change Canada, Montreal, Canada; 4 McGill Bird Observatory, Montreal, Canada; 5 Disney’s Animals, Science and Environment, Orlando, FL USA; 6 Purple Martin Conservation Association, Erie, PA USA; 7 Consolidated Nuclear Security, LLC, U.S. Department of Energy-National Nuclear Security Administration Pantex Plant, Amarillo, TX 79120 USA; 8 Present Address: 8500 Kemper Road, Canyon, TX USA; 9 Instituto Nacional de Pesquisas da Amazônia (INPA), Manaus, Brazil

**Keywords:** Aerial insectivore, Biologging, Foraging, Habitat selection, Home range, Songbird

## Abstract

**Background:**

“Central-place foragers” are constrained in their habitat selection and foraging range by the frequency with which they need to return to a central place. For example, chick-rearing songbirds that must feed their offspring hourly might be expected to have smaller foraging ranges compared to non-breeding songbirds that return nightly to a roost.

**Methods:**

We used GPS units to compare the foraging behaviour of an aerial insectivorous bird, the purple martin (*Progne subis*), during the breeding season in three regions across North America, as well as the non-breeding season in South America. Specifically, we tested foraging range size and habitat selection.

**Results:**

Foraging range did not vary among regions during breeding (14.0 ± 39.2 km^2^) and was larger during the nonbreeding period (8840 ± 8150 km^2^). Purple martins strongly preferred aquatic habitats to other available habitats year-round and in the Amazon commuted from night roosts in low productivity sediment-poor water, where risk of predation was probably low, to daytime foraging sites in productive sediment-rich water sites.

**Conclusions:**

We provide the first estimates for foraging range size in purple martins and demonstrate foraging preference for aquatic habitats throughout two stages of the annual cycle. Understanding foraging constraints and habitat of aerial insectivores may help plan conservation actions throughout their annual cycle. Future research should quantify foraging behaviour during the post-breeding period and during migration.

**Supplementary Information:**

The online version contains supplementary material available at 10.1186/s40462-022-00337-2.

## Background

Many animals return to a central place when foraging, such as a roost, nest or perch [1]. For these so-called “central-place foragers”, foraging time includes transit time to and from the central place in addition to searching and handling time, and ‘optimal’ foragers are expected to select nearby foraging patches and travel along the most direct path to and from the central place [1, 2]. Distant foraging patches will be used only if net energy gain is higher than at nearby patches [3–6]. The frequency at which the central place is visited will, in part, determine the average foraging range and, consequently, may influence habitat selectivity (i.e. time spent per patch and selectivity of patch quality) necessary to optimize energy gain [7]. Within a species, individuals that must return frequently to a central place, such as a nest, would be expected to have smaller foraging ranges and be less selective than individuals that only need to return to a central place once per day, such as a nightly roost.

Foraging behaviour of migratory birds varies throughout the year as they use different environments during different parts of their annual cycle: breeding, migratory stopovers, and wintering. Northern saw-whet owl (*Aegolius acadius*) males have larger home ranges in winter than in spring and overlap with other individuals only during winter [8]. Habitat preference in eastern whip-poor-wills (*Antrostomus vociferus*), which are aerial insectivores, foraging on flying insects while in flight [9], differs throughout the annual cycle: forests in winter and open habitats during breeding [10]. Other aerial insectivores, such as swallows that often roost in large congregations, must be able to adjust their foraging behaviour to environmental variation throughout the annual cycle, including movement of the central place. Birds that nest in small colonies during the breeding season and roost in large groups during winter are central-place foragers that will be constrained by the frequency of return to the central place [11–13]. Because breeding birds must feed their offspring many times per day, but outside of breeding need only return to a roost once per day, foraging range may be much larger during the non-breeding than breeding season.

Habitat selectivity could increase, decrease or be similar between the breeding and non-breeding season. Assuming that the distance between patches of similar quality increases with patch quality (i.e. low quality patches are closer together than high quality patches because high quality patches are rare), habitat selectivity could increase with foraging range because patch quality increases with transit time and, so, individuals traveling farther are selecting rarer, more heterogeneous habitats [14–16]. Thus, non-breeding birds that have greater flexibility may take advantage of farther, high-quality patches, increasing selectivity. Moreover, if individuals deplete patches more readily when they make many trips to nearby patches (i.e. breeding), then they may select nearby habitats as the high quality patches are depleted if they are highly constrained, as is the case for chick-rearing birds [17]. Alternatively, habitat selectivity may be similar regardless of foraging range if there is no scale-dependence in patch quality and no prey depletion, and breeding season habitat selection may be representative of non-breeding habitat selection, as is the case in penguins [18].

However, it is also possible that more energetically constrained breeding birds with smaller foraging ranges would be more selective of habitat compared to less constrained non-breeding birds with larger foraging ranges. Habitat composition and quality may differ throughout the year, influencing foraging range size and habitat selection. Chick-rearing birds must feed their nestlings frequently and may target seasonally abundant food sources that are relatively close to the central place, allowing them to have small foraging ranges. If seasonally abundant food sources are patchy, birds should show strong habitat selection. If seasonally abundant food sources are uniformly distributed in the landscape, however, birds can have small foraging ranges without showing strong habitat selection [14–16].

We used the purple martin (*Progne subis*) as a model species for colonial aerial insectivores because they have central nests or roosts at throughout most of the year and they are one of the largest swallows, allowing them to be tracked using recently-miniaturized GPS biologgers [19, 20]. Given that aerial insectivores are a rapidly declining avian guild [21–23], and there is no consensus on the main cause of decline [9, 24, 25], understanding their year-round habitat requirements is critical for developing management practices that would contribute to conservation [26, 27]. Purple martins breed in North America and winter in South America, where individuals from different breeding sites mix in enormous roosts, some containing over 10,000 birds [20, 28–31]. Similar to other aerial insectivores, purple martins have declined by 30% in North America over the last 5 decades [32]. They feed on flying insects, some of which are associated with water, such as dragonflies [33]. Non-breeding roosts are in island-type habitats close to bodies of water and, in the Amazon basin, in flooded forest near streams, rivers, and other wetlands [20, 34]. In the Amazon, large differences in nutrient availability can be observed between different water types [35], which may affect the foraging behaviour of purple martins. Although their roosting and nesting habitat is well described across the purple martin’s range, foraging habitat has not been well studied.

We deployed GPS units on adult purple martins to collect data during chick-rearing and the over-wintering period to examine habitat selection and foraging range, providing the first year-round, fine-scale information on the foraging behaviour of a small neotropical migrant. We hypothesized purple martins would be constrained year-round by central-place foraging and predicted that (1) foraging range will be smaller and habitat selectivity weaker in chick-rearing than in overwintering birds, (2) purple martins select resources in water-based habitats because they are often thought to catch and consume insects with aquatic stages, and (3) purple martins will preferentially forage in nutrient-rich habitats in the Amazon. We used goodness of fit (pseudo-R^2^) to assess habitat selectivity; if individuals are selecting particular habitats then the pseudo-R^2^ would be higher, and otherwise it would be lower. While central place foraging is a well-established concept [1, 2], few studies test it in the field.

## Methodology

### Data collection

Fieldwork for this study was carried out from 2016 to 2020 across 4 regions: Quebec (Canada), Pennsylvania (USA), Florida (USA), and Texas (USA). Nests were monitored approximately twice per week to follow breeding success. Chick-rearing adult purple martins were captured in their nest boxes using either (1) a trap door on the outside of the compartment that could be manually lowered when the individual went in its nest compartment, (2) a lightweight trap door propped up by a stick inside the nest compartment that would be tripped as a bird entered the compartment, or (3) a paint roller attached to a long pole that researchers used to block the entrance hole when a bird entered the compartment. We used targeted trapping to avoid causing unnecessary stress to non-target individuals [36]. A total of 100 GPS tracks (Lotek PinPoint10 or Pathtrack, ~ 1 g) were obtained from individual purple martins between 2016 and 2020. We attached GPS units using leg-loop harnesses [37]. The combination of GPS and harness weighed no more than 3% of an individual’s body mass [38]. In Quebec, GPS tags (Lotek PinPoint10, ~ 1 g) placed on breeding birds were programmed to take points every 10 min beginning approximately 30 min before sunrise, collecting data for about one day until the battery ran out, with 17 GPS tracks obtained from 21 deployments in 2020. In Orlando, Florida, we obtained 73 GPS tracks (81% recovery, Lotek PinPoint10, ~ 1 g) from chick-rearing birds in 2016, 2017, and 2018 across 7 colonies. GPS units were programmed to take points beginning 30 min before sunrise for either 1-min or 10-min intervals and collected data for approximately 1 day each. In Texas, 8 GPS units (Lotek PinPoint10, ~ 1 g), were deployed on chick-rearing individuals in 2020 with 4 points recorded per day, 3 during the day and 1 at night, and two units were retrieved. GPS data for each track spanned 10–15 days. Non-breeding tracks from 8 individuals (Pathtrack nanoFix GEO-MINI or Lotek PinPoint10, ~ 1 g, 2018–2020) were deployed in the breeding season prior to migration and recaptured on return to southern Quebec (Canada, 9% recapture rate), Orlando (Florida, 81% recapture rate, USA), Erie (Pennsylvania, USA, 17% recapture rate), or Amarillo (Texas, USA, 25.0% recapture rate) the following year. Non-breeding tags were programmed to take points two to four times per day, typically with one point at night to confirm roost location, and GPS data spanned weeks or months.

### Foraging range

We analyzed foraging range of every GPS track, using colony or roost as a central place. Distance from the colony or roost was calculated for every point. Points were labeled as foraging if they were > 100 m from the colony (to exclude points where breeding birds were presumably carrying food to and from their nests) and if they were taken during the day; non-foraging points were discarded. Non-breeding roosts in South America were studied indirectly using GPS data. They were identified by manually screening GPS fixes during the night to detect points where birds were found repeatedly. Eleven roosts were identified from 8 GPS tracks. Because some martin roosts are quite large, overnight points within ~ 500 m of each other were considered the same roost. Daytime points between consecutive nights at the same roost were assigned to that roost. If a bird changed roosting locations, the daytime points between two different roosts were not assigned to a roost. We estimated foraging range for each individual using the ctmm package [39] in R. This package calculates home ranges using continuous-time stochastic processes and selects the best model based on maximum likelihood fitting and AIC selection [39]. The ctmm package accounts for autocorrelation among points, sampling frequency, and number of points [39], though we discarded any individuals with fewer than 10 foraging points. To examine predictors of foraging range and area, we used linear mixed models with foraging range as the dependent variable and breeding status (chick-rearing or non-breeding), latitude, and year as independent variables. We used the R packages lme4 [40] and lmerTest [41], and considered breeding status (categorical), latitude, and the interaction between latitude and breeding status as fixed effects and year as a random effect. In our study, we use *p* = 0.05 as the threshold for statistical significance.

### Habitat selection analysis

**Fig. 1:**
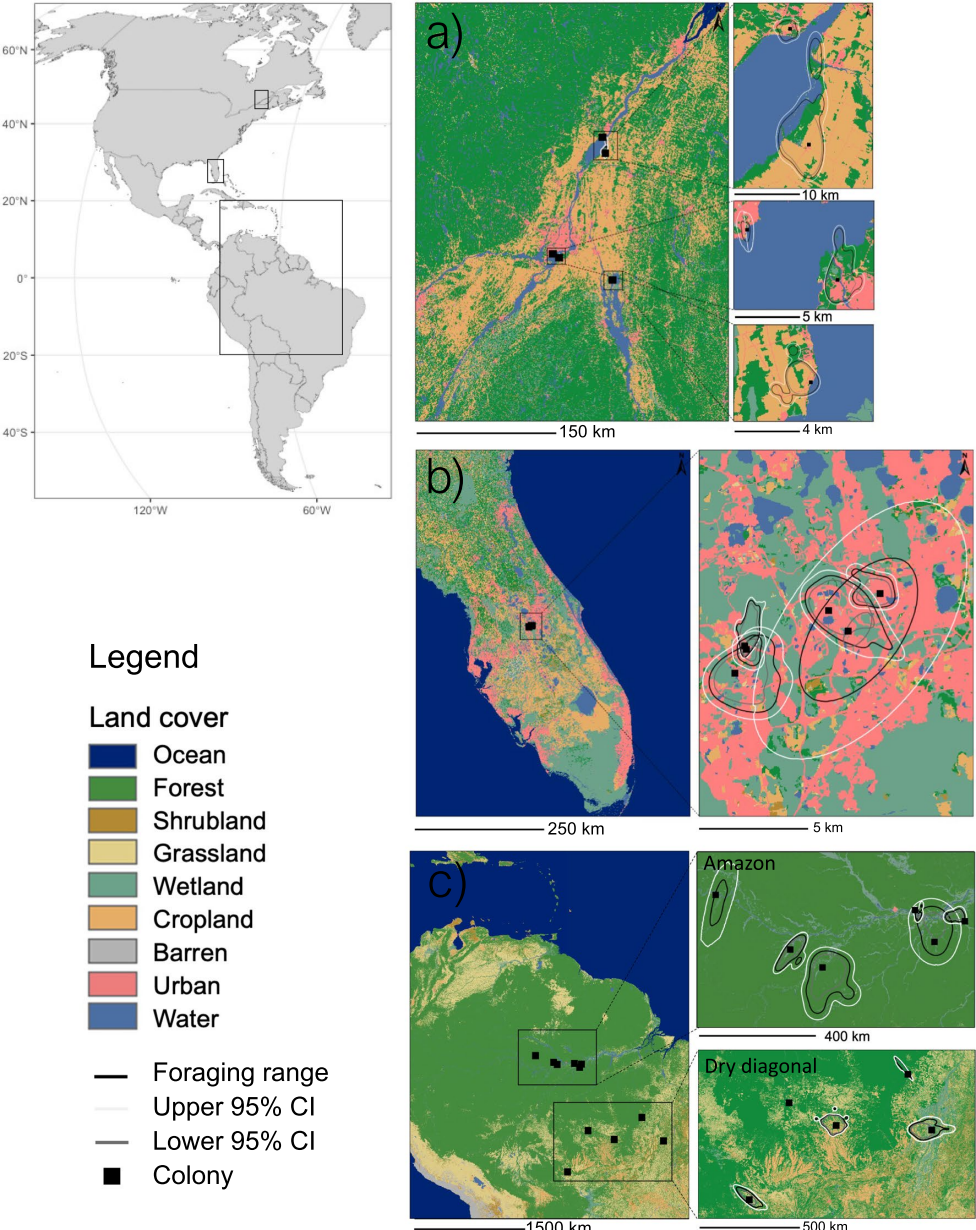
Representative foraging range estimates for each colony or roost with confidence intervals showing habitat types for **a** Quebec, **b** Florida, and **c** the Amazon and dry diagonal in South America. To the right of each lettered figure are details of areas indicated by the rectangles in each

We analysed habitat selection in four regions: Quebec, Florida, the Amazon, and “dry diagonal” (*cerrado* habitat in central South America, Fig. 1). We treated the Amazon and dry diagonal separately as they differ in habitat composition (Fig. 1) and, additionally, nutrient levels in water differ greatly and are linked to differential productivity [35]. We did not have sufficient data to analyze foraging habitat selection for Texas. For habitat selection analysis, we generated two sets of random points to be able to compare habitat selection at different scales: one based on the distance distribution (exponential) of each individual’s GPS data relative to the central point and another based on twice the distance distribution of an individual’s foraging points. This allowed us to examine habitat selection at two different scales. We did not examine larger scales because this would have meant including habitats outside of the ecoregion of interest of some individuals (non-breeding) or beyond the foraging radius of some individuals (breeding). We used the best available landcover data: 30 m Landsat and RapidEye raster land cover data for North America from the Commission for Environmental Cooperation (NALCMS, [42], 2015) and 100 m land cover data derived from Copernicus Global Land Operations ([43], 2020) for South America. We simplified land cover data; for example, we aggregated forest types into a single category. Wetland was defined as land cover where vegetation is adapted to live in soils saturated by water throughout the year or at least seasonally, including marshes, swamps, bogs, and, particularly in the Amazon, seasonally flooded forest. We defined open water as land cover consisting of freshwater bodies including rivers and lakes; purple martins did not use saltwater bodies in our study.

To evaluate habitat quality in the Amazon, we extracted nearest water sediment value for purple martins in the Amazon using a raster of river sediment values for the Amazon basin as a proxy for productivity [35, 44]. For both GPS points and randomly generated locations, we created 100 m, 200 m, and 500 m buffers around each point for breeding birds and 250 m, 500 m, and 1000 m for non-breeding birds and generated habitat selection models for each Quebec (breeding), Florida (breeding), Amazon (non-breeding), and dry diagonal (non-breeding) comparing used vs random habitats. Because purple martins move quickly while foraging, we used buffers around each point for all points, including the actual GPS foraging points and randomly generated 1x and 2x points based on distance distribution, to give greater context to the landscape where purple martins were actively foraging, rather than to simply extract land cover from points. The minimum buffers were determined based on the grain of the remote sensing information. We used larger buffer sizes for the South America land cover dataset (Copernicus, 100 m) compared to the North American dataset (NALCMS, 30 m) as the Copernicus dataset has a larger grain. Moreover, birds foraging over larger areas are likely assessing habitat at a larger grain size than those foraging over smaller areas, so this also made sense biologically; based on visual inspection of the data, non-breeding birds foraged over greater distances. For each wetland and open water, we calculated the length of edge between each of those habitat types and adjacent habitat types within the buffers surrounding points and refer to each of those as “wetland edge” and “water edge”, respectively.

In our resource selection models, we used colony as a random effect for breeding birds and ID for non-breeding birds. We added distance from the central place [16] as well as year as a fixed effect for regions that had more than 1 year; all had 3 years or less. In the Amazon, we added nearest water sediment value as a fixed effect to account for productivity [35, 44]. We carried out logistic regression using the “glmer” function in the R package lme4 and selected buffer size using AIC selection. We created correlation matrices for each region to evaluate correlation between habitat types. For each region, we removed strongly correlated habitat types (i.e., > 0.7) from the models to reduce multicollinearity. For each set of 1x and 2x distance distributions, we compared AIC values of full models at different buffer sizes to select optimal buffer size for each region. Then, we compared different model types: full, water-based, natural, open, and null models for each region. We calculated pseudo-R^2^ as a proxy to measure strength of habitat selection.

## Results

Chick-rearing purple martins averaged a maximum foraging distance across individuals of 2.94 km ± 2.26 km (N = 92) from the colony whereas non-breeding birds travelled much farther from their roost, averaging 78.4 km ± 37.9 km per roost (N = 11, Table 1, t_100_ = 5.61, *p* < 0.01, Additional file 1: Table S1). There was non-significant evidence of variation in foraging area among breeding regions controlling for the effect of year and colony (F_2,81_ = 2.99, *p* = 0.06). Non-breeding birds overwintering in central South America also had much larger home ranges than chick-rearing birds (t_8_ = 5.62, *p* < 0.01), and we found no evidence that latitude predicted foraging range size (t_2_ = − 1.85, *p* = 0.25) when controlling for the random effect of year (Fig. 1, Additional file 1: Table S2).

**Table 1:** Foraging range area (± SD) and maximum foraging range by region for purple martins

	Region	N	Foraging area (km^2^)			Maximum foraging distance (km)		
			Mean	Min.	Max.	Mean	Min.	Max.
Breeding	Quebec	17	7.19 ± 14.6	0.44	63.1	2.68 ± 3.04	0.66	14.0
	Texas	2	11.1 ± 9.05	4.73	17.5	4.18 ± 2.94	2.10	6.26
	Florida	73	15.7 ± 43.3	0.33	362	2.97 ± 2.05	0.51	10.7
Non-breeding	Amazon	6	9980 ± 9970	669	26,400	78.0 ± 39.9	30.3	148
	Dry diagonal	5	7470 ± 6130	209	14,900	77.7 ± 39.9	11.9	117

For breeding birds, N is the number of individuals, while N is the number of roosts for non-breeding birds

For our habitat selection analyses in Florida, correlation matrices revealed that wetland and developed habitats (built-up human-dominated environments) were strongly correlated (R^2^ =  − 0.90 1x, R^2^ =  − 0.86 2x model); thus, developed habitat was removed from the habitat selection analysis as we believed it to be less biologically relevant. Furthermore, for both the Amazon and dry diagonal, forest was strongly correlated with other habitat types and was removed (Amazon: forest and water R^2^ =  − 0.83 1x, R^2^ =  − 0.77 2x; dry diagonal: forest and grassland, shrubland, cropland R^2^ =  − 0.90 1x, R^2^ =  − 0.92 2x), and wetland and wetland edge (Amazon: R^2^ = 0.74 1x, R^2^ = 0.73 2x; dry diagonal: R^2^ = 0.82 1x, R^2^ = 0.89 2x) and open water and water’s edge (Amazon: R^2^ = 0.42 1x, R^2^ = 0.46 2x; dry diagonal: R^2^ = 0.74 1x, R^2^ = 0.77 2x) were strongly correlated, and we removed the edge habitats from the model. For Quebec, the 50 m buffer size ranked best while the 200 m buffer size ranked best for Florida based on AIC. The 1000 m buffer Amazon model ranked the best and we chose the 1000 m buffer size for the dry diagonal as the 500 m and 1000 m models were within AIC = 0.2 of each other to allow us to better compare the non-breeding models (Additional file 1: Table S3). For all regions, the full model ranked best based on AIC (Additional file 1: Table S4).

In Quebec, purple martins selected water edge (1x distance z = 5.66, *p* < 0.001; 2x distance z = 5.91, *p* < 0.001) while avoiding wetland edge at both scales (1x distance z =  − 2.57, *p* = 0.01; 2x distance, z =  − 2.33, *p* = 0.02, Table 2). Other habitats were used proportionally to their availability, and therefore were neither selected nor avoided (i.e., *p* > 0.05, Table 2). Pseudo-R^2^ increased from the 1x to 2x scale (0.10 and 0.46, respectively).

**Table 2:** Model outputs for Quebec habitat selection with a 50 m buffer at a 1x distance distribution (R^2^ = 0.10) and 2x distance distribution (R^2^ = 0.46)

	1x distance				2x distance			
	Estimate	Std. error	z value	*p* value	Estimate	Std. error	z value	*p* value
(Intercept)	− 3.03	0.281	− **10.8**	**< 0.001**	− 0.788	0.345	− **2.29**	**0.02**
Barren, developed	12.9	22.2	0.582	0.6	15.3	21.3	0.716	0.5
Grassland, shrubland, cropland	12.4	22.2	0.559	0.6	14.8	21.3	0.692	0.5
Forest	12.9	22.2	0.58	0.6	15.2	21.3	0.713	0.5
Wetland	13.6	22.2	0.61	0.5	15.8	21.3	0.739	0.5
Open water	11.5	22.2	0.518	0.6	13.7	21.3	0.641	0.5
Wetland edge	− 0.00621	0.00242	− **2.57**	**0.01**	− 0.00568	0.00243	− **2.33**	**0.02**
Water edge	0.00741	0.00131	**5.66**	**< 0.001**	0.0076	0.00129	**5.91**	**< 0.001**
log(Distance to colony)	0.236	0.0429	**5.51**	**< 0.001**	− 0.156	0.0372	− **4.19**	**< 0.001**

z values in bold are significant (*p* < 0.05)

In Florida, purple martins selected wetlands (1x distance z = 8.96, *p* < 0.001; 2x distance z = 10.4, *p* < 0.001) and avoided forested habitat (1x distance z = -2.67, *p* = 0.008; 2x distance z =  − 2.88, 0.004) at both scales (Table 3). As we removed developed habitat due to its strong negative correlation with wetland, purple martins in turn should avoid developed areas as they selected wetlands. At the 2x scale, they avoided grassland, shrubland, and cropland habitats (z =  − 2.26, *p* = 0.024, Table 3). Open water, wetland edge, and water edge were neither selected nor avoided (i.e., *p* > 0.05). Pseudo-R^2^ increased with scale (1x, R^2^ = 0.03; 2x, R^2^ = 0.06), but the explanatory power of both models is low.

**Table 3:** Florida best model outputs with a 200 m buffer at 1x distance distribution (R^2^ = 0.03) and 2x distance distribution (R^2^ = 0.06). Models exclude urban because of its high (negative) correlation with wetland

	1x distance				2x distance			
	Estimate	Std. error	z value	*p* value	Estimate	Std. error	z value	*p* value
(Intercept)	− 2.41	0.134	− **18**	**< 0.001**	− 0.0191	0.122	− 0.157	0.88
Grassland, shrubland, cropland	− 0.386	0.21	− 1.84	0.07	− 0.458	0.203	− **2.26**	**0.024**
Forest	− 1.364	0.512	− **2.67**	**0.008**	− 1.44	0.5	− **2.88**	**0.004**
Wetland	0.65	0.0727	**8.96**	**< 0.001**	0.723	0.0692	**10.4**	**< 0.001**
Open water	− 0.477	0.447	− 1.07	0.29	− 0.384	0.393	− 0.978	0.33
Wetland edge	0.000313	0.00058	0.54	0.59	0.000876	0.00059	1.49	0.14
Water edge	0.00169	0.0017	0.992	0.32	0.000381	0.00164	0.232	0.82
log(Distance to colony)	0.128	0.019	**6.71**	**< 0.001**	− 0.237	0.0171	− **13.8**	**< 0.001**
Year2017	− 0.0188	0.0685	− 0.274	0.78	− 0.0467	0.053	− 0.882	0.38
Year2018	− 0.0462	0.0633	− 0.731	0.46	0.0594	0.0559	1.06	0.3

z values in bold are significant (*p* < 0.05)

In the Amazon, martins selected wetland (1x distance z = 7.68, *p* < 0.001; 2x distance z = 7.12, *p* < 0.001) and habitats near high-sediment (nutrient-rich) water compared to low-sediment water (1x distance z = 2.51, *p* = 0.01; 2x distance z = 4.23, *p* < 0.001) and avoided open water (1x distance z =  − 3.46, *p* < 0.01; 2x distance z =  − 3.45, *p* < 0.001) at both scales (Table 4). Grassland, cropland, shrubland and developed/barren habitat were neither preferentially selected nor avoided (i.e., *p* > 0.05, Table 4). Pseudo-R^2^ slightly decreased between the 1x (R^2^ = 0.26) and 2x (R^2^ = 0.20) scales but was low overall.

**Table 4:** Model output for the Amazon with a 1000 m buffer for 1x distance distribution (R^2^ = 0.26) and 2x distance distribution (R^2^ = 0.20) removing forest, wetland edge, and water edge with 1000 m buffer

	1x distance				2x distance			
	Estimate	Std. error	z value	*p* value	Estimate	Std. error	z value	*p* value
(Intercept)	− 3.47	0.997	− **3.44**	**< 0.001**	1.4	0.85	1.65	0.1
Grassland, shrubland, cropland	− 3.34	2.54	− 1.32	0.2	− 3.88	2.38	− 1.63	0.1
Barren, developed	1.96	1.31	1.49	0.1	1.93	1.58	1.23	0.2
Open water	− 2.38	0.688	− **3.46**	**< 0.001**	− 2.19	0.632	− **3.45**	**< 0.001**
Wetland	4.03	0.525	**7.68**	**< 0.001**	3.22	0.452	**7.12**	**< 0.001**
Nearest water sediment	0.0184	0.00733	**2.51**	**0.01**	0.0333	0.00789	**4.23**	**< 0.001**
log(Distance to roost)	0.161	0.1	1.61	0.1	− 0.318	0.083	− **3.83**	**< 0.001**
Year2019	− 0.115	0.274	− 0.418	0.7	0.0889	0.265	0.335	0.7
Year2020	0.131	0.375	0.35	0.7	0.338	0.375	0.9	0.4

z values in bold are significant (*p* < 0.05)

In the dry diagonal south of the Amazon, purple martins selected open water habitats (z = 2.56, *p* = 0.01) and avoided barren and developed habitat (z =  − 0.519, *p* < 0.001) as well as grassland, shrubland, and cropland habitat (z − 3.28, *p* < 0.001) at the 1x scale. Wetland was neither selected nor avoided (*p* = 0.5). At the 2x scale, purple martins avoided grassland, shrubland, and cropland habitat (z =  − 2.10, *p* = 0.04), while wetland, open water, and barren and developed habitat were neither selected nor avoided (i.e., *p* > 0.05) (Table 5). Pseudo-R^2^ was small for both scales: 0.07 for the 1x distribution and 0.08 for the 2x distribution.

**Table 5:** Model output for the dry diagonal with a 1000 m buffer for 1x distance distribution (R^2^ = 0.07) and 2x distance distribution (R^2^ = 0.08) removing forest, wetland edge, and water edge

	1x distance				2x distance			
	Estimate	Std. error	z value	*p* value	Estimate	Std. error	z value	*p* value
(Intercept)	− 4.74	0.7	− **6.77**	**< 0.001**	− 0.209	0.634	− 0.329	0.7
Grassland, shrubland, cropland	− 0.775	0.236	− **3.28**	**0.001**	− 0.49	0.233	− **2.10**	**0.04**
Barren, developed	− 2.65	5.11	− **0.519**	**< 0.001**	− 10	6.29	− 1.59	0.1
Open water	1.71	0.666	**2.56**	**0.01**	1.28	0.772	1.66	0.1
Wetland	0.7	0.999	0.697	0.5	1.028	1.25	0.822	0.4
log(Distance to roost)	0.332	0.0722	**4.6**	**< 0.001**	− 0.144	0.0637	− **2.27**	**0.02**
Year2020	− 0.292	0.183	− 1.6	0.1	0.0396	0.177	0.223	0.8

z values in bold are significant (*p* < 0.05)

In all regions except the Amazon, habitat selectivity was higher at the 2x scale than at the 1x scale, and it was higher in the Amazon than in all other regions using pseudo-R^2^ measures of selectivity at the 1x scale (Additional file 1: Table S5).

## Discussion

As expected, the frequency of return to the central place (nest or roost) is important in predicting foraging range size, with foraging area roughly 100 times larger during the non-breeding period. Thus, the movement of adult birds while chick-rearing appears to be constrained by the need to feed their chicks frequently and to defend their nests against conspecific competitors and nest predators [11]. In contrast, non-breeding purple martins, which return to the roost only once per day and are not territorial, and therefore have greater flexibility to travel further, foraged up to 117 km from the roost. Purple martins are likely not territorial while feeding across their range and often feed together, taking advantage of local food sources and insect emergences [45]. Breeding birds could be taking advantage of seasonal resource pulses associated with higher latitudes which may reduce foraging range size; however, we did not find evidence for a latitudinal gradient across regions and colonies in foraging range size. We found that non-breeding purple martins had larger foraging ranges compared to breeding purple martins. These non-breeding birds could be foraging further away from the central place to take advantage of patchy resources [14, 15] and targeting areas with higher productivity (i.e., selected habitats at or near high water sediment), which may partially explain their larger foraging ranges. This first test of central foraging theory across the range of a long-distance migratory songbird shows the importance of the constraint imposed by frequency of return to the central place on foraging throughout the year.

Whether or not a species is a central place forager in various stages of the annual cycle affects foraging range size at different times of the year. The differences in foraging range size for a given species across different stages in the annual cycle are greater for central place foragers than territorial species. For example, territorial migratory songbirds such as ovenbirds (*Seiurus aurocapilla*) and black-and-white warblers (*Mniotilta varia*) show similar territory sizes during breeding and non-breeding [46–51], whereas our purple martins had much larger foraging ranges during the non-breeding period compared to breeding. Since many aerial insectivores, particularly swallows and including purple martins, roost in large numbers during winter, our results are likely applicable to many aerial insectivores. The foraging ranges we estimate here are the first we are aware of that have been documented for purple martins, and are generally larger than those measured for other aerial insectivores both in terms of distance from the colony or roost and foraging area. For example, chick-rearing bank swallows (*Riparia riparia*) had foraging ranges that were less than 2 km [12]; chick-rearing tree swallows (*Tachycineta bicolor*) had foraging ranges of “a few hundred metres” [52]; overwintering eastern whip-poor-wills (*Antrostomus vociferus*) had mean home ranges of 0.0524 km^2^ ± 0.0054 km^2^ [10]; and home ranges of breeding European nightjars (*Caprimulgus europaeus*) were 0.24–0.40 km^2^ [53]. Purple martins tend to fly higher on average while foraging (mean 119 m [54], 162 m [33]) compared to other swallows such as tree swallows (mean 69 m) and barn swallows (mean 34 m) [54]), which could partially explain their larger foraging ranges. Furthermore, purple martins are the largest swallows in North America [33] and their larger foraging ranges relative to smaller swallows could also be related to greater body mass [55].

Purple martins in our study generally selected wetland habitat, open water, and water edge habitats. Purple martins in the Amazon tended to roost near water with low sediment, which is nutrient-poor, but foraged over or near high-sediment water, which is rich in nutrients [56]. This pattern may result from purple martins taking advantage of patchy resources; where they traveled from sediment-poor or low-productivity roosts at night that may offer greater protection from predators but travel to more productive, or sediment-rich, foraging sites during the day. Sediment-poor water is associated with lower abundance of fauna in general [56–59] and so is associated with less prey but also fewer predators compared to high-sediment water. Sediment-rich water has higher insect prey abundance, but more predators [56–61]. Thus, it follows that sediment-poor sites are safer for roosting, and sediment-rich sites are better for foraging. Predators to purple martins across their range include aerial and terrestrial animals, such as raptors [24]. The individuals that spent time in the dry diagonal also spent time in the Amazon as purple martins overwinter in the Amazon or pass through it during migration if they overwinter further south [20]. There are a few biases in our habitat selection analysis: in some areas, wetland is a seasonal habitat especially in the Amazon where water levels fluctuate widely throughout the year and forest is flooded (i.e., considered wetland) during certain parts of the year and therefore productivity may fluctuate throughout the year [44]. Hence, seasonality may affect actual land cover compared to the land cover data we use. Furthermore, sediment values and therefore productivity may also be affected by time of year as water levels change [44].

Non-breeding purple martins are less constrained than breeding individuals in terms of foraging range size (2.94 km for breeding vs. 78.4 km for overwintering), only returning to a central place once per day. Since chick-rearing birds need to feed their nestlings frequently and therefore have a higher energy balance, they must forage closer to the central place and are more constrained in their foraging range. Because non-breeding purple martins are only returning to a central location once per day and are only feeding themselves, they can travel greater distances and can move from nutrient-poor to nutrient-rich habitats to meet their needs during different times of the day. In their non-breeding habitat in South America and especially in the Amazon, forest cover was the dominant land cover type, yet it was not selected; relatively unconstrained purple martins were easily able to forage over their preferred habitats.

For Quebec, Florida, and the dry diagonal, habitat selection strengthened as the scale of the random points increased. The Amazon had the strongest 1x-scale habitat selection, likely because of large differences in prey abundance between high- and low-sediment waters. Considering the relatively small distances from the colony travelled by breeding birds, low habitat selectivity at the 1x scale suggests that purple martins choose nest locations and roosts in good quality habitats. Because aquatic emergent insects could be spilling over into different habitats, patches may be relatively uniform surrounding the colony. The large increase in model fit (pseudo-R^2^) observed in Quebec (R^2^ = 10 to 46%) was greater than the other regions and could indicate that there are limited areas where habitat is suitable for breeding in Quebec where purple martins are declining more strongly than in other regions [32]. As such, habitat may be more patchy at a large scale, and purple martins may be selecting colonies near water to take advantage of insect emergences from the water to provision their young (e.g., mayflies, personal observation). Many of the colonies in Quebec had a high proportion or availability of agricultural lands or developed/urban areas, which were neither selected nor avoided, and may not be high-quality foraging habitat for purple martins, potentially acting as sinks. The strength of habitat selection tended to be greater at the 2x scale among region, except for the Amazon, although absolute values tended to be small. For Quebec and Florida, the strength of habitat selection showed a stronger increase with scale compared to the Amazon and dry diagonal, possibly because foraging ranges on purple martins’ overwintering grounds are very large compared to their breeding foraging ranges and the effect of scale is less important for large foraging ranges. Overall, we did not observe strong trends in the strength of habitat selection between breeding and non-breeding purple martins; regional differences in habitat composition and food abundance likely affect the strength of habitat selection [62], and purple martins show similar preferences for habitat throughout their annual cycle, similar to a habitat selection study in penguins [18]. Furthermore, colony size for breeding birds is generally smaller than non-breeding roost size (personal observation). Larger foraging ranges in overwintering versus breeding purple martins could be due in part to “Ashmole’s halo”, where a higher density of roosting birds in South America compared to breeding colonies in North America could suggest that birds in the Amazon may need to travel further to find foraging areas because of prey depletion near the roost [63]. However, we do not have information for roost size for all the non-breeding roosts, and birds in the Amazon travelling from roosting habitat near low-sediment water to more suitable high-sediment foraging habitat may also partially explain the larger foraging ranges observed in South America.

Because purple martins forage on flying insects above ground or water, they are likely less constrained by fine-scale habitat type than ground- or tree-foraging species, especially in winter when they can travel large distances during the day. Since purple martins are foraging above habitats rather than within them, there may be a spillover effect of insects with larval stages in aquatic habitats that fly above other habitats after emergence, as purple martins frequently eat insect prey with aquatic stages [33, 64, 65]. Birds in our study tended to nest near bodies of water or wetland and non-breeding birds spent a lot of their time within a few kilometers of rivers or wetland. Water-type habitats are therefore important in their annual cycle, and future conservation planning should protect water-based habitats, especially wetlands, including preserving or improving water quality. Other migratory swallows might show similar trends in foraging range across the annual cycle and may also require large amounts of land for overwintering.

Future studies could examine repeatability in foraging range and habitat selection within individuals. We assumed that points away from the central place during the day represented foraging points; however, some of these points may represent perching or commuting rather than foraging, and that is a limitation of our study. Another limitation of our study is that we lack data on insect availability. Incorporating insect availability could allow for patchiness and habitat quality to be better evaluated and allow for optimal foraging theory to be further tested. Future work could aim to coarsely identify foraging trips in GPS tracks sampled at 1-min or 10-min intervals, for example using hidden Markov models. If technology advances sufficiently, GPS-accelerometers and GPS-altimeters could be deployed on purple martins to identify bouts of active foraging in three dimensions and narrow habitat selection analysis to these periods. Future studies could also examine foraging range and habitat selection of incubating, post-breeding, and migratory birds and assess the potential effects of climate and weather. Furthermore, work could be done to further examine the relationship between low-sediment and high-sediment waters on roosting and foraging behaviour, and if roost size plays a role in foraging range (i.e., Ashmole’s halo).

## Conclusion

Our study is the first to quantify purple martin foraging range and foraging habitat selection and test central place foraging theory across two points in the annual cycle. Purple martins have larger foraging ranges during the overwintering period compared to breeding, likely because chick-rearing birds are constrained by the need to return to the central place more frequently than non-breeding birds that return to a roost once per day, and they select water-related habitats across their range, indicating that freshwater aquatic habitats are important for the conservation of this declining species. Purple martins can forage hundreds of meters in altitude, and awareness of the importance of these aerial habitats year-round to aerial insectivores highlights the need for conservation practices that incorporate airspaces above and surrounding important habitats, in addition to the key habitats themselves that we identify here, particularly freshwater aquatic habitats [33, 66, 67].

## Supplementary Information

**Additional file 1:** Supplementary tables.

## Data Availability

A subset of data files supporting this paper are available on Movebank (ID 1910442974, https://www.movebank.org/cms/webapp?gwt_fragment=page=studies,path=study1910442974). Other files can be made available on request.
