# Supplementary material for: Central-place foraging poses variable constraints year-round in a neotropical migrant
Source: Mov Ecol. 2022 Sep 20;10:39. doi: 10.1186/s40462-022-00337-2 (PMC9487155; doi:10.1186/s40462-022-00337-2)
Supplement: Supplementary file 1 — Additional file 1: Supplementary tables. [file 40462_2022_337_MOESM1_ESM.docx]

Supplementary Table 1: Linear foraging range predicted by the fixed effects of latitude, breeding status, and the interaction of latitude and breeding status.

|  | Estimate | Std error | df | t value | p value |
| --- | --- | --- | --- | --- | --- |
| Intercept | 8.11 | 0.344 | 100 | 23.6 | <0.001 |
| Latitude | -0.0109 | 0.0107 | 100 | -1.02 | 0.308 |
| Breeding status | 3.00 | 0.534 | 100 | 5.61 | <0.001 |
| Latitude * breeding status | 0.0108 | 0.0488 | 100 | 0.221 | 0.826 |

Supplementary Table 2: Foraging range area predicted by the fixed effects of latitude, breeding status, and the interaction of latitude and breeding status.

|  | Estimate | Std error | df | t value | p value |
| --- | --- | --- | --- | --- | --- |
| Intercept | 2.91 | 0.639 | 1.25 | 4.55 | 0.100 |
| Latitude | -0.0365 | 0.0197 | 1.48 | -1.85 | 0.248 |
| Breeding status | 5.46 | 0.972 | 7.88 | 5.62 | <0.001 |
| Latitude * breeding status | 0.0242 | 0.0883 | 27.3 | 0.274 | 0.786 |

Supplementary Table 3: AIC rankings of models with different buffer sizes. Bolded AIC values represent selected models.

| Non-breeding | Dry diagonal | 2x | AIC | 924 | 919 | **916** |
| --- | --- | --- | --- | --- | --- | --- |
|  |  |  | df | 8 | 8 | 8 |
|  |  | 1x | AIC | 914 | 908 | **908** |
|  |  |  | df | 8 | 8 | 8 |
|  | Amazon | 2x | AIC | 682 | 675 | **667** |
|  |  |  | df | 10 | 10 | 10 |
|  |  | 1x | AIC | 674 | 664 | **657** |
|  |  |  | df | 10 | 10 | 10 |
|  |  |  | Buffer (m) | 250 | 500 | 1000 |
|  | Florida | 2x | AIC | 14663 | 14628 | **14628** |
| Breeding |  |  | df | 11 | 11 | 11 |
|  |  | 1x | AIC | 14839 | 14814 | **14807** |
|  |  |  | df | 11 | 11 | 11 |
|  | Quebec | 2x | AIC | **2782** | 2792 | 2784 |
|  |  |  | df | 10 | 10 | 10 |
|  |  | 1x | AIC | **2826** | 2841 | 2844 |
|  |  |  | df | 10 | 10 | 10 |
|  |  |  | Buffer (m) | 50 | 100 | 200 |

Supplementary Table 4: AIC rankings of different habitat type models. Bolded AIC values represent selected model.

| Non-breeding | Dry diagonal | 1000m | 2x distance | AIC | **916** | 921 | 920 | 917 | 921 |
| --- | --- | --- | --- | --- | --- | --- | --- | --- | --- |
|  |  |  |  | df | 8 | 6 | 5 | 6 | 4 |
|  |  |  | 1x distance | AIC | **908** | 915 | 917 | 911 | 922 |
|  |  |  |  | df | 8 | 6 | 5 | 6 | 4 |
|  | Amazon | 1000m | 2x distance | AIC | **667** | 668 | 741 | 745 | 745 |
|  |  |  |  | df | 10 | 8 | 6 | 7 | 5 |
|  |  |  | 1x distance | AIC | **657** | 657 | 730 | 738 | 736 |
|  |  |  |  | df | 10 | 8 | 6 | 7 | 5 |
| Breeding | Florida | 200m | 2x distance | AIC | **14628** | 14639 | 14745 | 14701 | 14794 |
|  |  |  |  | df | 11 | 9 | 7 | 6 | 5 |
|  |  |  | 1x distance | AIC | **14807** | 14815 | 14892 | 14859 | 14932 |
|  |  |  |  | df | 11 | 9 | 7 | 6 | 5 |
|  | Quebec | 50m | 2x distance | AIC | **2782** | 2795 | 2851 | 2845 | 2850 |
|  |  |  |  | df | 10 | 7 | 5 | 4 | 3 |
|  |  |  | 1x distance | AIC | **2826** | 2831 | 2875 | 2868 | 2874 |
|  |  |  |  | df | 10 | 7 | 5 | 4 | 3 |

Table 5: Pseudo R^2^ for selected models

|  | Quebec | | Florida | | Amazon | | Dry diagonal | |
| --- | --- | --- | --- | --- | --- | --- | --- | --- |
|  | 1x | 2x | 1x | 2x | 1x | 2x | 1x | 2x |
| R^2^ | 0.10 | 0.46 | 0.03 | 0.06 | 0.26 | 0.20 | 0.07 | 0.08 |
| Nagelkerke’s R^2^ | 0.03 | 0.04 | 0.01 | 0.02 | 0.17 | 0.17 | 0.04 | 0.02 |
| McFadden’s R^2^ | 0.02 | 0.03 | 0.01 | 0.01 | 0.12 | 0.12 | 0.02 | 0.01 |
| Cox and Snell R^2^ | 0.02 | 0.03 | 0.01 | 0.01 | 0.10 | 0.10 | 0.02 | 0.01 |
